# Dental Nomenclature and Terminology

**Published:** 1879-04

**Authors:** J. Taft


### DENTAL NOMENCLATURE AND TERMINOLOGY.

BY J. TAFT.

A word is an utterance of the human voice which expresses a thought or a thing.

Language is the medium for communication of thought from one person to another.

This communication is perfect in proportion, as there is precision and definiteness in the language used, and a correct and uniform understanding of its meaning. The modifications of words should not be left subject to the whims and caprices of the pedant and charlatan. All words, and especially names and terms, should be defined and established upon such a basis as will give them the greatest range of usefulness, and the most enduring stability.

The misuse of words produces confusion of thought and ideas, and impairs the value of language as a medium of communication.

Perhaps there never was in the history of the human race a greater tendency nor a stronger effort to change, mutilate, adulterate and dilute a language than now exists in respect to the English languge.

There are many reasons for this. The English language has largely incorporated with and into it material from many other languages. The free admixture of different nationalities in this country is a prolific source of vitiation of pure English.

People of other languages abound with us. Their efforts to use our language results, as a rule, in a mongrel speech, very far from pure English. Their errors and incongruities are too often copied by those who should avoid them. Education has become popular, and a large proportion of the American people wish to appear, at least, to be cultured and educated; the reality is, however, often much below the assumption, so that the misuse and abuse of language greatly abounds.

Many are in the habit of using words and phrases, the true meaning and power of which they have little or no conception, even in the grosser sense.

The half way or would be scientist is often guilty of the most absurd use of names, terms and phrases.

The press pours forth a ceaseless current of literature, incalculable in amount; and the preparation of this matter is, to a large extent, in the hands of those unfitted for the work.

The free indulgence in slang words and phrases, and provincialsms, tends greatly to the corruption of language. Loose and careless habit in the use of language weakens its power, and lessens its efficiency.

The Anglo-Saxon, with its immediate descendant, the modern English, is said to "have the strength of iron, with the gleam and sparkling of burnished steel." "It is the *mother tongue* of the English language, about four-fifths of the words in actual use being from this source. Not only in the number of words, but in their peculiar character and importance, as well as their influence on grammatical forms, Anglo-Saxon constitutes its principal strength.

"At the same time that our chief peculiarities of structure and idiom are essentially Anglo-Saxon, from the same copious fountain have sprung words designating the greater part of objects of sense, the terms which most frequently occur in discourse, and which recall the most vivid conceptions, as *sun*, *moon*, *earth*, *fire*, *day*, *night*, etc., and words expressive of the dearest connections, the strongest and most powerful feelings of nature, from our earliest days, as *mother*, *father*, *sister*, *brother*, *wife*, *home*, etc.

The language of business, of the shop, the market, and of every day life, our national proverbs, our language of humor, satire and coloquial pleasantry; the most energetic words we can employ, whether of kindness or invective, in short, words expressive of our strongest emotions and actions, in all the most stirring scenes of life, from the cradle to the grave, are derived from the Anglo-Saxon."

Every speaker or writer then, who would not only convince the understanding but teach the affections, should adopt Anglo-Saxon expressions, which from early use and dearest associations excite emotion, and affect the heart.

Every department of human occupation has its peculiar and distinctive names, words and phrases. In some they are far more numerous and prominent than in others. This is true in a remarkable degree in the callings denominated professions, for instance the Law, Theology and Medicine. And perhaps in none do distinctive names and phrases so abound as in the latter.

There seems to be a necessity for these, growing out of the multiplicity of parts, and their relations to each other, and their reciprocal influence. Should these designations be abandoned, we can hardly conceive how endless and inextricable confusion could be avoided. In medical literature a large proportion of the distinctive names are taken unchanged from other languages, and chiefly from the *Greek* and *Latin*—finished languages, so far as the form and construction is concerned—sometimes, falsely called, "*dead languages.*" How can a language be denominated dead, that enters so largely into the constitution of the leading languages of the world.

TO BE CONTINUED.

